# Impact of health talks on knowledge, attitudes and perception of breast cancer screening and treatment amongst healthcare staff by a breast surgical unit in a public healthcare institution: a cross-sectional study

**DOI:** 10.1186/s12905-021-01424-z

**Published:** 2021-08-21

**Authors:** Jeffrey Jun Xian Hing, Wai Peng Lee, Yen Nee Sophia Chua, Pei Ting Tan, Chi Wei Mok, Spoorthi Shetty Sudhakar, Chin Mui Seah, Su-Ming Tan

**Affiliations:** 1 Division of Breast Surgery, Department of General Surgery, Changi General Hospital, Singapore, Singapore; 2 Department of Nursing, Changi General Hospital, Singapore, Singapore; 3 Clinical Trials and Research Unit, Changi General Hospital, Singapore, Singapore; 4 Singhealth-Duke NUS Breast Centre, Singapore, Singapore

**Keywords:** Breast cancer awareness, Screening, Knowledge, Perception

## Abstract

**Background:**

In October 2019, surgeons from Changi General Hospital (CGH) Breast Centre delivered a series of health talk for its employees to assess the knowledge and perception of breast cancer screening and to improve the level of related knowledge amongst the institution’s healthcare workers. This was to enable CGH, a healthcare provider to not only care for our patients, but also to look after its staff.

**Methods:**

141 hospital staff attended a 40-min talk followed by an open question and answer forum. Pre and post talk surveys were conducted to gauge knowledge, attitudes, beliefs and misconceptions towards breast cancer screening and treatment. Question domains were divided into (1) breast cancer knowledge, (2) breast cancer screening guidelines and (3) attitudes and perception of breast cancer screening and treatment. Univariate and multivariate logistic regression analysis were used to examine the relationship between demographics and performance in question domains.

**Results:**

The overall response rate was 131 out of a total of 141 attendees (92.9%). The median age was 44 years old (range, 22–67), with nursing staff making up 40% of the cohort. Analysis showed statistically significant improvement in median score across all 3 domains. (*p* < 0.05) after the forum. We found that respondents who were women ≥ 40 years (eligible age for screening), had higher income, lived in larger housing types, had attended previous talks, had served > 10 years in healthcare and had personal encounter with breast cancer patients performed better. Surprisingly, being a nurse or having a university degree did not translate to a better score. 99% of respondents found the forum beneficial and would recommend it to others. Several knowledge gaps about breast cancer screening and misconceptions were identified. Future campaigns should focus on raising awareness of the national screening program BreastScreen Singapore. We aim to reinforce its recommendations, promote on the affordability and ready accessibility.

**Conclusions:**

A simple Breast Cancer Awareness Month campaign targeted at healthcare workers was found to be effective at educating hospital staff on breast cancer, screening practices and improving perception of screening and treatment practices. This may empower them to not only care for themselves but also to serve patients better.

## Background

Breast cancer is the most common and lethal cancer amongst women in Singapore, accounting for 17.3% or 2105 deaths of all cancer in women in Singapore from 2011 to 2015 [1]. Moreover, the age standardised incidence rate^1^ of breast cancer had jumped more than 2.5 times over the last four decades from 24.6 to 65.3 per 100,000 population per year [1]. After the Singapore Breast Screening Project concluded in 1994, the national breast cancer screening programme- BreastScreen Singapore (BSS) was launched in 2002. Despite considerable efforts made to encourage women to go for screening, uptake rates remained low [2, 3]. Regular campaign activities such as talks conducted during the Breast Cancer Awareness Month (BCAM) in October were targeted to promote awareness, so as to increase screening uptake.

Healthcare workers served as a direct source of medical information to the public and patients. Due to their frequent contact with patients and public, they were often looked upon to provide accurate health related information. It was therefore essential, that they conveyed accurate facts when promoting health awareness amongst the population. There were reports on observations whereby healthcare staff including nurses, radiographers or physiotherapists or patient service associates may not necessarily have the basic knowledge of these relevant topics [2, 4].

In October 2019, breast surgeons at Changi General Hospital (CGH), a public healthcare institution in the Eastern part of Singapore, delivered a series of talks on breast cancer and mammographic screening to its employees. We took the opportunity to assess the participants’ knowledge, perception and practice with regards to breast cancer screening before and after the talk. The aim of this study was to determine the effectiveness of the breast cancer awareness talks at identifying areas of deficient knowledge amongst healthcare workers, clarifying common misconceptions on breast screening and improving perception towards breast cancer treatment.

## Methods

This cross-sectional study involved a convenient sampling of a target population attending the annual Breast cancer awareness campaign conducted hospital-wide. In October 2019, three health talks were conducted by CGH breast surgeons for staff members. Invitations were extended to all staff via email, intranet announcement and posters within the hospital premise. The content covered included epidemiology, signs and symptoms, risk factors of breast cancer, breast cancer screening guidelines, costs and accessibility to screening services. Common myths and misconceptions regarding breast cancer, screening and treatment were also addressed. All three talks had the same content but were conducted on different days in order to allow more staff to attend.

A self-administered questionnaire in English was conducted before and after the talk (Appendix 1). This questionnaire was adapted from a previous study looking at knowledge and perception of breast screening in Singapore [4]. A validity test was not performed on the questionnaire. The questionnaire was designed to assess three domains—knowledge of breast cancer, knowledge of breast cancer screening in Singapore and attitudes and perception towards breast cancer screening and treatment. The questions and answers were listed in Appendices 1 and 2. The list of accepted answers was not exhaustive and answers with similar implied meaning were accepted. Feedback with regards to the talk (duration, usefulness and content) was also obtained.

Participation of the questionnaires was strictly voluntary and anonymous. An immediate response was requested, and the questionnaires were collected upon completion. Participants were encouraged not to discuss the answers with each other. Entries were checked by study assistants to ensure completeness of response to maintain a high fidelity and response rate. The study protocol has been reviewed and approved by the Singhealth Centralised Institutional Review Board (CIRB Ref no. 2019/ 2829).

The data including, total correct responses percentages before and after the health talk, was analysed with Chi-square test. We calculated pre and post questionnaire median scores and chose a 75th percentile as cut off score to analyse the group of better performing respondents according to their demographic characteristics. We determined the correlation of variables to predict for better scores. Multivariate analysis was performed on the statistically significant variables, using logistic regression analysis, with the Statistical Package for the Social Sciences (SPSS) version 18.0 (SPSS Inc, Chicago, IL, USA). A *P* value of < 0.05 was taken to be statistically significant.

## Results

### Demographics

The overall response rate was 92.9% (131 out of a total of 141 attendees). The median age of the respondents was 44 years old (range: 22–67). Chinese participants formed 50.4% of our respondents. The majority (97.9%) of respondents were female, nurses (40.4%) and were diploma/degree holders (85.1%) (Table 1).

**Table 1:** Respondents’ demographics, working experience and exposure to family, friends and/or patients with breast cancer (n = 141)

Demographics	Respondents (%)
Age*	
< 40	88 (62.4)
40–49	27 (19.1)
≥ 50	26 (18.4)
*Ethnic group*	
Chinese	71 (50.4)
Malay	35 (24.8)
Indian	9 (6.4)
Eurasian	2 (1.4)
Others	24 (17.0)
*Sex*	
Male	3 (2.1)
Female	138 (97.9)
*Highest education completed*	
Primary or below	0 (0.0)
Secondary	13 (9.2)
Diploma (Polytechnic, A level, ITE)	28 (19.9)
University degree holder	92 (65.2)
Missing	8 (5.7)
*Healthcare profession*	
Doctor	0 (0.0)
Nurse	57 (40.4)
Allied health	36 (25.5)
Administrative staff/others	48 (34)
*Gross personal monthly income (SGD)*	
< $2000	9 (6.4)
$2000—$3999	64 (45.4)
$4000—$5999	49 (34.8)
$6000 and above	13 (9.2)
*Housing*	
Rental/1–2 room apartment	11 (7.8)
3 Room apartment	16 (11.3)
4–5 Room/executive apartment	92 (65.2)
Executive condominium	11 (7/8
Landed property	2 (1.4)
Missing	9 (6.4)
*Family history of breast cancer*	
Yes	16 (11.3)
No	113 (80.1)
Don’t know	4 (2.8)
Missing	8 (5.7)
*Know of relative who have breast cancer*	
Yes	52 (36.9)
No	70 (49.6)
Don’t know	11 (7.8)
Missing	8 (5.7)
*Know of friends who have breast cancer*	
Yes	63 (44.7)
No	64 (45.4)
Don’t know	6 (4.3)
Missing	8 (5.7)
*Had previously and/or currently been involved in the care of breast cancer patients*	
Yes	42 (29.8)
No	90 (63.8)
Missing	9 (6.4)
*Attended previous similar health talk*	
No	97 (68.8)
Yes	43 (30.5)
Missing	1 (0.7)
*Duration in healthcare*	
≤ 10 years	75 (53.2)
> 10 years	64 (45.4)
Missing	2 (1.4)

^*^Age group categorised according to BreastScreen Singapore guidelines[5]

< 40: Not recommended for screening mammograms

40–49: To consider annual mammograms

≥ 50: Recommended for biennual screening mammograms

### Working experience and exposure to family, friends and/or patients with breast cancer

Slightly more than half (53.2%) had been working in healthcare for 10 years or less with two-thirds (68.8%) not having attended similar talks. Most did not have a family history of breast cancer (80.1%) and had not been involved in the care of breast cancer patients (63.8%) (Table 1).

### Domain 1: knowledge of breast cancer

The improvement in knowledge scores pre- and post-talk was statistically significant in 16 out of 27 questions as detailed in Table 2 (*P* < 0.05). The main areas of pre-talk knowledge deficit (which was defined as having less than 50% of the cohort having the correct responses) were: signs and symptoms of breast cancer, risk factors, and the stages of breast cancer. These were indicated with asterisks in Table 2.

**Table 2:** Number (%) of respondents achieving the correct answer pre and post talk (n = 131)

	Pre (%)	Post (%)	% Increase	*P* value
*Knowledge of breast cancer*				
1. What is the most common cancer in Singapore women	105 (80.2)	128 (97.7)	27.5	< 0.001
2. Is breast cancer a common cancer among Singapore women	124 (94.7)	130 (99.2)	4.5	0.031
3. Name two risk factors for breast cancer*	28 (21.4)	96 (73.3)	51.9	< 0.001
4. Name two symptoms of breast cancer*	59 (45.0)	82 (62.6)	17.6	0.001
*Breast cancer has different stages as follows*				
1. Stage 0*	44 (33.6)	122 (93.1)	59.5	0.001
2. Stage 1	123 (93.9)	129 (98.5)	4.6	0.109
3. Stage 2	127 (96.9)	128 (97.7)	0.8	1.000
4. Stage 3	123 (93.9)	127 (96.9)	3.0	0.388
5. Stage 4	117 (89.3)	126 (96.2)	6.9	0.035
*Treatment of breast cancer may involve*				
1. Traditional medicine	92 (70.2)	99 (75.6)	5.4	0.265
2. Surgery	128 (97.7)	131(100.0)	2.3	NA
3. Chemotherapy	128 (97.7)	130 (99.2)	1.5	0.625
4. Radiotherapy	115 (87.8)	129 (98.5)	10.7	0.001
*Knowledge of breast cancer screening in Singapore*				
1. Have you heard of the BreastScreen Singapore program	73 (55.7)	127 (96.9)	41.2	< 0.001
2. Correctly identify what is considered to be “Breast Cancer Screening”*	49 (37.4)	59 (45.0)	7.6	0.132
3. Correctly identify age at which women are to start going for breast screening	92 (70.2)	122 (93.1)	22.9	< 0.001
4. Correctly identify the interval for breast screening *	47 (35.9)	102 (77.9)	42.0	< 0.001
5. Correctly name 2 places that screening mammograms are available (specific) *	19 (14.5)	69 (52.7)	38.2	< 0.001
6. Correctly identify how much a screening mammogram costs at the BSS screening centre for Singaporeans? *	35 (26.7)	115 (87.8)	61.1	< 0.001
7. Can Medisave^+^ be used to pay for screening mammogram for women ≥ 50 years old?	77 (58.8)	87 (66.4)	7.6	0.174
8. How often women should do breast self-examination (BSE)?	85 (64.9)	115 (87.8)	22.9	< 0.001
*Attitudes and perceptions of Breast cancer screening and treatment*				
1. Women should only go for a mammogram when they have a breast problem eg lump	112 (85.5)	116 (88.5)	3.0	0.535
2. I think that regular screening mammogram will not affect the shape of the breasts	114 (87.0)	127 (96.9)	9.9	0.004
3. If there is an abnormality in the breast, the 1st thing I would do or advise others to do is to see a doctor	121 (92.4)	130 (99.2)	6.8	0.004
4. Will there be a higher chance of cure if breast cancer is detected early?	127 (96.9)	130 (99.2)	2.3	0.375
5. Surgery for breast cancer always needs the whole breast to be removed	105 (80.2)	115 (87.8)	7.6	0.052
6. Surgery for breast cancer will cause the affected arm to swell and be crippled	86 (65.6)	111 (84.7)	19.1	< 0.001

^*^Weaker areas defined as less than 50% of cohort scored correctly

+ MediSave is a compulsory national saving scheme in Singapore

After the talk, there was significant improvement in scores of more than 50% absolute increase in correct respondents in 3 questions, namely risk factors of breast cancer, stages of breast cancer, and the costs of screening mammograms for Singaporeans. 41.2% more respondents were aware of BSS and 42.0% more respondents knew the correct interval of screening mammograms after the talk.

However, post-talk, 24.4% of the respondents still believed that traditional medicine was part of treatment for breast cancer.

### Domain 2: knowledge of breast cancer screening

Generally, there was poor knowledge of breast screening amongst the respondents. Prior to the talk, almost half (44.3%) have not heard of BSS. Most (62.6%) were unaware that screening mammogram was to be done when there are no breast symptoms or problems and 85.5% were unable to correctly name two locations for screening mammograms. Almost two-thirds (64.1%) did not know the recommended interval for routine screening mammograms. Majority (72.8%) did not know the costs of a screening mammogram, with more than 50% (n = 74) believed that it cost more than SGD$100, when in fact, it cost SGD$50 when done at the BSS screening centres.

After the talk, the respondents displayed a significant improvement (*p* < 0.05) in most areas. Although there was improvement in their understanding of what was considered to be screening mammogram (37.4–45.0%) and the knowledge that Medisave (a national compulsory saving scheme) could be used to subsidise the screening mammograms (58.8–66.4%), it did not reach statistical significance [6].

Despite a significant improvement, 47.3% of the respondents were still unable to accurately name two locations to perform screening mammogram and 22.1% were unaware of the correct interval for screening mammogram.

### Domain 3: attitudes and perception of breast cancer screening and treatment

Most of the respondents (> 80%) provided correct answers to the questions in this domain, except for the question on the effect of breast cancer surgery on the ipsilateral arm. Fortunately, there was significant improvement after the talk, from 65.6 to 84.7% (*p* < 0.05).

In terms of personal practice, only 31.5% (34/108) of eligible respondents had gone for screening mammogram. After the talk, 82% of the respondents reported that they would go for screening mammogram and most (99%) would recommend their family and friends to go for screening too.

### Scores

**Fig. 1:**
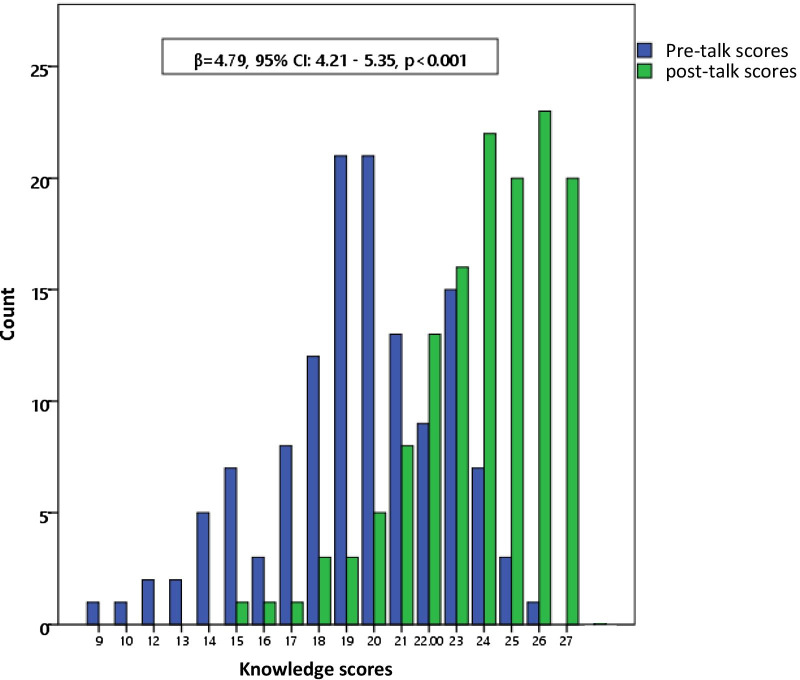
Analysis of knowledge scores pre and post talk

The total score for all three domains was 27. The mean total score pre-talk was 19 (range: 9–26). There was a significant improvement of the post-talk mean score to 24 (range: 15–27) (*p* < 0.05) (Fig. 1). Using the 75th percentile as cut-off, a high score on knowledge was arbitrarily defined as those with a score of > 21 (22/27 = 81.5%) and a low score was defined as those with a score of ≤ 21. There was a significant improvement in the percentage of overall good performers and a consistent improvement in mean scores across all 3 domains (Table 3). Of the three domains, respondents fared worst in the knowledge of breast cancer screening.

**Table 3:** Proportion (%) of poor and good performers, breakdown of mean scores in all 3 domains pre and post-talk

		Pre-talk (%)		Post-talk (%)	
Poor performers (scoring ≤ 21/27)		n = 96 (73.3)		n = 22 (16.8)	
Good performers (scoring > 21/27)		n = 35 (26.8)		n = 109 (83.2)	
Average scores		19 (range: 9–26)		24 (range:15–27)	
Domains	Total score	Average score among poor performers (%)	Average score among good performers (%)	Average score among poor performers (%)	Average score among good performers (%)
1. Knowledge of breast cancer	13	9.54 (73.4)	11.34 (87.2)	10.27 (79.0)	12.12 (93.2)
2. Knowledge of breast cancer screening	8	3.72 (46.5)	6.09 (76.1)	4.41 (73.5)	7.04 (88.0)
3. Attitudes and perceptions of Breast cancer screening and treatment	6	4.01 (66.8)	4.80 (80.0)	5.07 (84.5)	5.56 (92.7)

The scores were analysed against the demographic, working experience and exposure to family, friends and/or patients with breast cancer of the respondents. We found that respondents who were women ≥ 40 years (eligible age for screening), had higher income, lived in larger housing types, had attended previous talks, had served > 10 years in healthcare and had personal encounter with breast cancer patients performed better. Surprisingly, being a nurse or having a university degree did not translate to better score (Table 4). Having a non-nursing background and longer duration of service were the two statistically significant variables that predicted for better scores in the pre-talk analysis.

**Table 4:** Multivariate logistics regression on pre-talk knowledge scores

	Knowledge		Univariate		Multivariate	
	Poor (%)	Good (%)	OR (95%CI)	*P* value	OR (95%CI)	*P* value
*Age*						
< 40	67 (80.7)	16 (19.3)	REF		REF	
≥ 40	29 (60.4)	19 (39.6)	2.74 (1.24, 6.08)	0.013	1.67 (0.55, 5.08)	0.364
*Education*						
Non-university	25 (67.6)	12 (32.4)	REF		REF	
University	66 (75.9)	21 (24.1)	0.66 (0.29, 1.54)	0.341	1.73 (0.51, 5.91)	0.380
*Occupation*						
Non-nurse	54 (56.8)	22 (62.9)	REF		REF	
Nurse	41 (75.9)	13 (24.1)	0.78 (0.35, 1.73)	0.538	0.18 (0.05, 0.64)	0.008
*Personal income*						
< $4000	30 (83.3)	6 (16.7)	REF		REF	
≥ 4000	52 (66.7)	26 (33.3)	2.50 (0.92, 6.76)	0.071	1.45 (0.39, 5.42)	0.578
*Housing type*						
3 Room and smaller/rented	21 (84.0)	4 (16.0)	REF		REF	
4–5 Rooms	64 (73.6)	23 (26.4)	1.89 (0.59, 6.08)	0.288	0.74 (0.16, 3.29)	0.687
EC/private	6 (54.5)	5 (45.5)	4.38 (0.89, 21.61)	0.070	3.18 (0.36, 28.21)	0.298
*Know someone with breast cancer*						
No	26 (28.6)	5 (15.2)	REF		REF	
Yes	65 (71.4)	28 (84.8)	2.24 (0.78, 6.43)	0.134	1.81 (0.50, 6.56)	0.368
*Attended talk previously*						
No	69 (77.5)	20 (22.5)	REF		REF	
Yes	26 (63.4)	15 (36.6)	1.99 (0.89, 4.46)	0.095	1.65 (0.56, 4.88)	0.366
*Working in healthcare*						
≥ 10 years	59 (85.5)	10 (14.5)	REF		REF	
> 10 years	35 (58.3)	25 (41.7)	4.21 (1.81, 9.80)	0.001	4.76 (1.36, 16.62)	0.014

^*^Multivariate analysis included age, education level, occupation, personal income, housing type, family history, relative and/or friends with breast cancer, attended previous talk, duration of working in healthcare

We further analysed the nurses’ performance in the 3 domains and found that the mean score for knowledge on breast cancer and attitude and perception about breast cancer screening and treatment were higher than average at 11.1/13 (85.3%) and 5.2/6 (86.7%) respectively, but lower than average for knowledge on breast cancer screening at 4.7/8 (58.7%) (Table 3).

### Feedback

In general, the talks were well received, with 99% of respondents finding the talk beneficial and would recommend to others to attend. 89.4% identified health talks as their preferred source of information on breast cancer (Table 5).

**Table 5:** Respondents’ preferred medium for future health awareness campaigns (%)

Health talks/public forums	126 (89.4)
TV	96 (68.1)
Printed materials such as posters, brochures	91 (64.5)
Internet	77 (54.6)
Family doctors	50 (35.5)
Email	21 (14.9)
SMS	15 (10.6)
Health App	74 (52.5)

## Discussion

### Scores

Our study showed statistically significant improvement of knowledge scores before and after the BCAM talks in all three domains of questions (Fig. 1; Tables 2 and 3). The weakest performing domain was the domain on breast cancer screening. Fortunately, the post-talk questionnaire showed the improvement in knowledge of breast screening to be the greatest. This may be due to the fact that healthcare staff who worked in a tertiary hospital that was not a screening centre, were therefore not familiar with screening practices. Thus, this would be an area that future talks to healthcare staff should place more emphasis on.

The multivariate logistic regression revealed that working > 10 years in healthcare and non-nurses had better performance, suggesting that having a nursing background alone did not translate to better overall scores. Our subgroup analysis revealed that although nurses performed worst in the knowledge of breast cancer screening, but they had above average scores in knowledge of breast cancer, attitudes and perceptions towards treatment. This was similar to previous studies that showed that nurses in a tertiary hospital lacked knowledge in screening even though they are aware of the symptoms, risk factors, treatment and natural history of the breast cancer [7, 8]. Chong et al. [9] also reported that nurses in hospital setting fared worse than their counterparts in primary health settings in terms of breast screening knowledge. Heena et al. [10] also reported that the knowledge attitudes and practices related to breast cancer screening among healthcare professionals were found to be lower than expected.

The authors postulated that as CGH was a tertiary referral centre, nurses were more accustomed to managing more advanced breast conditions and hence lacked experience in promoting breast cancer screening practices. Another possible explanation could be that the nurses who attended the talk were a bias group. As a large number of nurses work shift duties, some may not be able to attend the talks. Hence those who attended may not be representative of the entire nursing population in the hospital. However, the authors took comfort in that, despite their initial poorer knowledge, their scores improved significantly after the talk.

### Knowledge gap

#### Domain 1: knowledge on breast cancer

Despite a significant improvement in knowledge of risk factors and symptoms of breast cancer post-talk, 26.7% and 37.4% of the respondents respectively were still unable to provide correct answers for these after the talk. This suggested that the talk was effective in improving knowledge, but perhaps in future talks, more emphasis could be placed in these specific areas.

Post-talk, 24.4% still held the belief in treatment for breast cancer may involve traditional medicine. These were areas that might have to be emphasized in future talks. This may be due to the deep-rooted cultural beliefs in traditional remedies in the predominantly Asian population in Singapore. A local study found that there was a wide discrepancy between the understanding and its uptake rate. The understanding of complementary and alternative medicine (CAM) may be poor among the population, but almost half of the surveyed population continued to use CAM concurrently with conventional medicine and up to 70% would not consult a doctor or pharmacist when using CAM [11]. Future talks should highlight the lack of scientific evidence on CAM in breast cancer treatment.

#### Domain 2: knowledge on breast cancer screening

This was the weakest performing domain before and after the talk. Despite significant post-talk improvement, 22.1% and 47.3% of the respondents were still unaware of the correct interval for screening mammogram and unable to accurately name two screening centres respectively. More than half (55.0%) had poor understanding of what was considered to be screening mammogram and a third (33.6%) did not know that Medisave could be used to pay for the screening mammogram.

This was alarming since BSS started in 2002 and the use of Medisave for screening practices was permitted in 2011. Medisave was a national saving scheme that could subsidise screening practices [12, 13] Poor screening attendance may in part be due to some of these knowledge gaps. This reinforced the need for continual efforts to educate and spread awareness of the national screening program to healthcare staff who could then help to educate the population. [14].

#### Domain 3: attitudes and perception of breast screening and cancer treatment

The respondents’ knowledge in this area was high except for the effect of breast cancer surgery on the ipsilateral arm. A higher proportion of respondents associated breast surgery with debilitating side effects. This misconception may be due to a higher probability of healthcare staff in a tertiary hospital encountering patients with more advanced stages of lymphoedema undergoing surveillance or rehabilitative processes.

#### Feedback

Majority of respondents’ feedback that their preferred way of obtaining health information was via health talks. The authors suggested that for future talks, emphasis could be placed in areas that were poor pre-talk (stage of breast cancer, BSS programme and breast cancer screening) with extra emphasis in areas that remained poor post-talk (risk factors, symptoms of breast cancer, traditional medicine, breast cancer screening). Future talks could also be tailored to the staff according to their area of work within the healthcare. For example, talks given to nurses, could have more prominence on information relating to breast cancer screening.

#### Limitations

We recognised that this study had a small sample size. In-person attendances suffered the constraint of venue capacity, hence limiting the number of attendees. To address this, more sessions and/or incorporating the talks into staff’s protected learning time could be arranged. Future talks may also be conducted online, thus allowing for even greater attendants. The study results were derived by convenient sampling. The instrument used to assess knowledge and perception in this case, although not validated, had been previously used in other studies to allow for convenient comparison [2, 4, 7]. This study was not designed to demonstrate that post-talk improvement in knowledge score would lead to an increase in screening practice.

Nonetheless, this study was able to highlight the knowledge gaps in breast cancer screening and treatment in healthcare staff. Majority of these areas showed significant improvement simply after attending the talk. This was a simple and effective way to vastly improve health knowledge amongst healthcare staff. As healthcare personnel are ambassadors of the healthcare system, their knowledge would aid in providing accurate information to patients and public, enabling them to make wise healthcare choices.

## Conclusions

This study had achieved its primary aim to identify certain knowledge gaps and perception towards breast cancer screening and treatment amongst healthcare staff. It also showed a significant post-talk improvement in knowledge scores, hence highlighting the effectiveness of such health talks. The authors endeavour to improve on future campaigns with targeted and relevant content.

## Data Availability

The datasets used and analysed during the current study are available from the corresponding author on reasonable request.

## Appendix 1: Questionnaire

Thank you for taking time to do this survey. We would like to obtain your feedback on the Breast Cancer Awareness Talk 2019. Please tick your answers in the boxes provided &/or fill in the blanks. Thank you!

This survey is anonymous and your personal details will not be disclosed.

## Abbreviations

CGH: Changi General Hospital
BSS: BreastScreen Singapore
BCAM: Breast cancer awareness month

## Footnotes

1 Age-standardised incidence (ASIR) is the rate that would be observed if the general population had the age structure of an external world standard population. Age standardisation facilitates the comparison of rates across time and also across countries.
